# Transportability of Conclusions From Confined Field Trials: A Case Study Using the Virus Resistant Transgenic Bean Developed in Brazil

**DOI:** 10.3389/fbioe.2020.00815

**Published:** 2020-07-21

**Authors:** Facundo Vesprini, Andrés Ignacio Maggi, Magdalena López Olaciregui, Natalia Andrea Módena

**Affiliations:** ^1^Biotechnology Directorate, Argentinian Ministry of Agriculture, Livestock and Fisheries, Buenos Aires, Argentina; ^2^ILSI Argentina, Working Group on Biotechnology, Buenos Aires, Argentina; ^3^National Service for Agrifood Health and Quality (SENASA), Buenos Aires, Argentina; ^4^Corteva Agriscience, Buenos Aires, Argentina; ^5^Bayer Crop Science, Buenos Aires, Argentina

**Keywords:** confined field trial, risk assessment, transportability of conclusions, transgenic bean, comparative studies, food/feed risk assessment, environmental risk assessment, criteria

## Abstract

The conceptual framework for Data Transportability, builds on the premise that well-designed studies conducted for the environmental and food/feed risk assessment of transgenic crops may be transportable across geographies. Beyond individual data, provided that certain criteria are met, the general conclusions of comparative assessments of a transgenic crop with its conventional counterpart would also be transportable. In spite of this, many regulatory agencies still require in-country field trials to complete risk assessments of transgenic crops. A sub-team from ILSI Argentina’s (International Life Sciences Institute, Argentina. www.ilsi.org.ar) Biotechnology Working Group tested the applicability of the transportability concept to the case of the *golden mosaic virus*-resistant transgenic bean, developed by EMBRAPA (EMBRAPA: Brazilian Agricultural Research Corporation). To this end, regulatory confined field trials (CFTs) carried out in Brazil to gather agro-phenotypic and compositional data were analyzed. The transportability of the conclusions of these studies to the bean cropping areas in Argentina was assessed as a conceptual exercise (with no intention to conclude on the biosafety of the common bean event). Comparative studies included the transgenic bean and its conventional parental line and were run in different agroecological environments so that any relevant differences could be observed. The main criteria to enable transportability were set by the sub-team and found to be met by the CFTs carried out in Brazil to inform a potential risk evaluation for Argentina.

## Introduction

Risk assessment for transgenic crops (also known as genetically modified crops) is typically conducted on a case-by-case basis using a weight-of-evidence approach. “Risk assessment based on an adequate problem formulation definition enables the development of plausible risk hypotheses that can be tested in order to identify and characterize risks” (Wolt et al., 2010). To assess risk, these hypotheses are tested using scientifically relevant information, which can derive from multiple sources, including field data. Results from well-designed studies conducted in laboratory, greenhouse, or in the field used for ERAs in one geography, are relevant to other geographies for the evaluation of the same or related transgenic crops (Garcia-Alonso et al., 2014; Ahmad et al., 2016). Furthermore, if these studies meet certain criteria, their conclusions should also be transportable. However, many regulatory agencies still require in-country field trials to complete risk assessments of transgenic crops intended for cultivation and even for import.

The concept of data transportability – data generated in one country used for the assessment in another country – focuses on the methodological quality of the studies and on the familiarity with crops, traits and receiving environments. As described by Capalbo et al., 2020 familiarity refers to the body of knowledge (evidence/data) and experience (of use, but also with risk assessment) with technologies and products that have undergone a risk assessment process or for which substantial data is available.

The Biotechnology Working Group from ILSI (International Life Science Institute) Argentina proposed to test the applicability of the concept of transportability to a real-world case. To this end, a sub-team was convened to investigate the transportability of conclusions from confined fields trials (CFTs) conducted in Brazil to Argentina, using as a case Embrapa 5.1, a transgenic common bean (*Phaseolus vulgaris*) resistant to the Bean Golden Mosaic Virus (BGMV) that was developed by EMBRAPA (Brazilian Agricultural Research Company).

The agro-phenotypic and compositional studies examined were based on a comparative field trial designed to measure biologically relevant differences between the transgenic crop and a conventional comparator for parameters that are informative for the risk assessment. Methodology and agronomic management of the studies, measured endpoints, and site selection, with focus on the diversity of tested environments were examined.

The exercise was limited to the applicability of the transportability concept, with no intention to conclude on the biosafety of the common bean event. This was a purely theoretical exercise as Embrapa 5.1 common bean is not under regulatory review in Argentina.

## Common Bean Production in Brazil and Argentina

**FIGURE 1:**
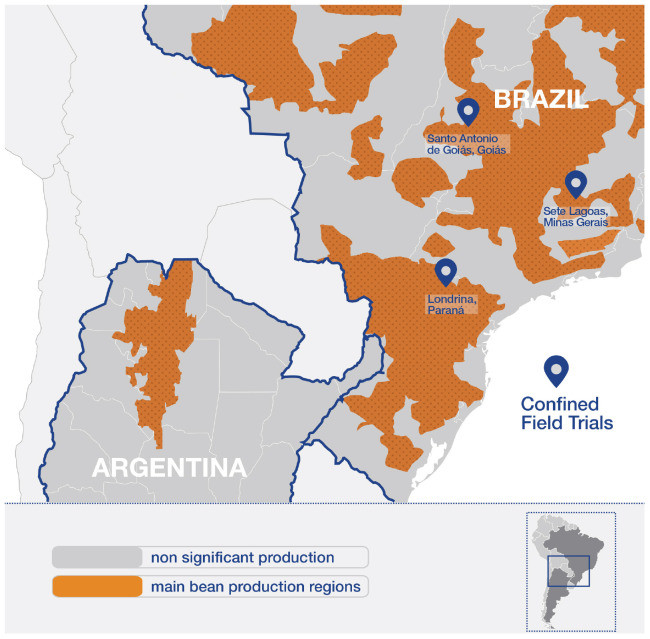
Main bean production regions in Brazil and Argentina, and locations where CFTs were performed.

*Phaseolus vulgaris* (common bean) is an annual species, native of Mesoamerica and South America, and its many varieties are grown worldwide for consumption (Alimentos Argentinos, 2016). Brazil is the main producer of common beans from the Mercosur region^1^, being also the main consumer, as common bean varieties are a basic component of the Brazilian diet, with an average production of 3 million tons per year. In Brazil, common bean is widely cultivated throughout the territory (Schoonhoven and Voysest, 1994). Main production regions are Paraná, Minas Gerais, Bahía, Goiás, and Mato Grosso (Clemente et al., 2017; Silva, 2019; Figure 1).

Argentina follows Brazil with 473.389 tons, with 95% of the production cultivated in the northwestern region of the country, at altitudes ranging from 300 to 1,000 m (Schoonhoven and Voysest, 1994; Alimentos Argentinos, 2016; Informe de Cadenas de Valor, 2016; Calzada and Treboux, 2019; Figure 1).

The main diseases of the common bean affecting productivity, are caused by fungi, bacteria, and viruses, like the BGMV, *Bean dwarf mosaic virus*, *Bean common mosaic virus*, and *Cowpea mild mottle virus* (Morales and Jones, 2004; Vizgarra et al., 2016). The BGMV disease was described for the first time in Brazil in the 1960s (Morales and Jones, 2004). This viral disease is transmitted by the whitefly *Bemisia tabaci* from wild species or legumes such as soybeans, that act as reservoirs for the virus (Vizgarra et al., 2016). When soybean is harvested the whitefly is forced to find alternative hosts; this time overlaps with the planting season of the common bean, which is in its most susceptible stage to viral infections (Vizgarra et al., 2016). Expansion of the soybean cultivation area resulted in an increase of whitefly populations, leading to a rapid dissemination of BGMV in the main bean-producing states of Brazil (Costa, 1975; Morales, 1981; Gálvez and Morales, 1994), and also in Argentina, where the first detections were reported in 1983 in the North West region (Vizgarra et al., 2012).

As reported, there is no bean variety in South America with an adequate level of resistance to this virus (Morales and Jones, 2004). Historically the control of the BGMV has become dependent on cultural practices including chemical control of the vector, physical distance from soybean fields, and the use of varieties with some degree of tolerance to viral infections (Vizgarra et al., 2006, 2016).

Within this context, EMBRAPA embarked in a project to develop a transgenic common bean line resistant to BGMV. This highly resistant line was named Embrapa 5.1 and was designed using a gene silencing approach. The inserted construct triggers post transcriptional gene silencing, by degradation of the *rep* gene mRNA, which is involved in functions that are necessary for viral replication. By silencing *rep* expression, the viral replication is compromised upon infection, resulting in plants resistant to the virus (Bonfim et al., 2007; Faria and Arãgao, 2013).

Brazil’s Technical National Commission on Biosafety (CTNBio) assessed the safety of Embrapa 5.1 for the environment and for human health, based on the studies submitted by EMBRAPA. As a result of this assessment, CTNBio approved Embrapa 5.1 for cultivation and consumption in 2011 (CTNBio, 2011).

All the information on Embrapa 5.1 reviewed during this exercise (section “Confined-Field Trials Conducted in Brazil for the Risk Assessment of Embrapa 5.1”), is publicly available at CTNBio’s website, as part of the dossier submitted to the regulatory agency (CTNBio, 2011).

## Considerations for Transportability of Conclusion

As Garcia-Alonso et al. (2014) described: “Field trials measurable endpoints vary, depending on the risk hypotheses being tested, but most of these studies are designed to identify differences between the transgenic crop and its non-transgenic counterpart, resulting from intended or unintended consequences of the genetic modification, across a range of agro-ecosystems.”

From the environmental perspective, a key study to identify these differences at the phenotypical level, is the agro-phenotypic study. The measurable endpoints in the CFTs that inform the study are crop specific and generally encompass those characteristics relevant to plant emergence, vegetative growth and those related to the reproductive biology of the plant.

From the food and feed safety assessment perspective, the concept of substantial equivalence provides a basis to determine if the foods/feeds derived from a transgenic plant are as safe as the conventional counterparts (OECD, 1993; FAO/WHO, 1996; Codex Alimentarius, 2003). Typical endpoints in compositional studies are key nutrients, antinutrients, secondary metabolites, and toxins for the transgenic crop and its comparator (FAO/WHO, 1991; OECD, 1993; WHO, 1995; Jonas et al., 1996). The samples for these compositional analyses are obtained from edible plant parts harvested from CFTs. Several documents provide a reference framework for the compositional assessment, facilitating the harmonization and transportability of these studies; among these, CODEX guidelines are the international standard (Codex Alimentarius, 2003). Additionally, OECD consensus documents on composition of crops, containing key nutrients, anti-nutrients and toxicants are widely used to identify the relevant components for a specific crop in a comparative analysis (OECD^2^). Also, there are other crop composition databases that provide baseline data and ranges of natural variability, established from multiple worldwide sources and seasons, for non-transgenic commercial cultivars (i.e., Agriculture & Food Systems Institute [AFSI], 2019).

As said, the comparative assessment between transgenic and non-transgenic crops involves plants grown side by side, that are therefore subject to the same environmental conditions and agronomic management. These are tested in different agro-climatic and agro-ecologic conditions within the crop production zones, under highly controlled conditions that will allow any biologically relevant differences arising from the gene insertion to be revealed.

CFT’s that are run in different environments are suitable to inform the risk assessment, regardless of the country/regions where they have been conducted, as long as they cover a range of different environmental conditions. Only if a specific risk hypothesis is identified for a particular receiving environment, that cannot be addressed by the available information, local CFTs might be required to generate new information. There is published evidence supporting transportability of data generated in different geographies for the ERA of transgenic soybean and maize (Horak et al., 2015; Nakai et al., 2015; Ahmad et al., 2016; Heredia Díaz et al., 2017; Corrales Madrid et al., 2018; Clawson et al., 2019; Matsushita et al., 2020). These publications show that, even when climate and production practices may be different, the environmental safety conclusions from the comparative assessments are consistent across geographies provided that studies are run across a broad range of conditions. Therefore, replicating field studies in every country or region where the transgenic crop is intended to be released would add new data, but would not change the conclusions reached in previous studies from other geographies.

To assess the transportability of conclusions from studies based on CFTs, the following criteria were proposed:

• Appropriate experimental design and methodologies.
• Relevance and consistency of measured endpoints across studies.
• Diversity of environmental conditions in CFTs’ locations within the crop production zones.

## Confined-Field Trials Conducted in Brazil for the Risk Assessment of Embrapa 5.1

Based on the information submitted by the developer of Embrapa 5.1, the sub-team focused on the agro-phenotypic and compositional studies to evaluate the transportability of their conclusions, as these are the most typically reviewed studies in the environmental and food/feed risk assessment processes, respectively. The sites selected to perform these studies were representative of common bean cultivation areas in Brazil, in three distinct regions, designated as Santo Antonio de Goiás, Goiás (GO), Londrina, Paraná (PR) and Sete Lagoas, Minas Gerais (MG) in 2008 and 2009 (see Figure 1).

### Assessment for Transportability of Conclusions

The sub-team applied the proposed criteria to assess the transportability of conclusions of these studies:

• Appropriate experimental design and methodologies:

Treatments included transgenic line Embrapa 5.1 and its conventional parent line (Olathe) as an appropriate comparator. Regarding the general agronomic management, cultural practices included: fertilization (after a soil analysis per site), irrigation, herbicide and insecticide applications. All these practices are typical for the bean production system. Likewise, the same crop management was uniformly applied across all plots at each site, helping to reduce the potential for non-trait related differences in pest pressure and agronomic performance among plots within a site. The study was conducted as a randomized complete block design, with 8 replicates per treatment at each location over 2 years. Data were analyzed by ANOVA using Statistical Analysis System (SAS) software to determine the effect of each treatment. Analyses were conducted across locations and years (location/year as a random factor) and for each location individually. Differences were considered significant at *P* < 0.05.

Regarding compositional studies, EMBRAPA developed a *de novo* common bean composition database, as a reference. To this end, eight common bean varieties were grown from 2003 to 2007 (5 years) in multiple locations. These reference materials provided a range of natural variation for each measured analyte. This database was later included in the OECD consensus document on compositional considerations for common bean (OECD, 2015).

• Relevance and consistency of the measured endpoints across studies:

The following characteristics were measured in the agro-phenotypic studies: yield, seedling emergence, seedling height, maximum width of the primary leaves, maximum length of the primary leaves, number of seeds per pod, weight of 100 seeds, pod length, pod width, seed length, seed width, thickness of seeds and flowering time. The sub-team considered that the selected parameters were appropriate and sufficient for risk characterization, as these adequately reflect the main phenotypic characteristics that are critical for productivity and common bean agronomic behavior.

In the compositional study, the endpoints considered for the analysis in raw and processed (cooked) beans included carbohydrates, vitamins B1 and B2, minerals, amino acids, and proximates. Anti-nutrients included phytic acid and trypsin inhibitors. These analytes are included in the recommendations of the OECD Consensus Document for common beans.

• Crop production areas where the CFTs were conducted:

As Faria and Arãgao (2013) mention, the edaphoclimatic conditions differed among locations. The sub-team reviewed the historical information on environmental factors (Instituto Nacional de Meteorología [INMET], 2020) for each location (GO, PR, and MG, Figure 1). Characteristics taken into account to assess the diversity of environmental conditions, included site locations (latitude / longitude), historical water balance, and environmental factors: temperature, humidity, and precipitation. Although soil type is not a key parameter for data transportability (Garcia-Alonso et al., 2014), it was also taken into account as secondary element to discriminate environments. The CFTs’ locations covered different bean production zones, and the evaluated characteristics taken together showed agronomically relevant differences between locations.

Based on this analysis of the three sets of criteria, the conclusions drawn from these studies were considered to be transportable for the purpose of a potential risk assessment in Argentina.

### Summary of Results and Conclusions Described in the Agro-Phenotypic and Compositional Studies

There were no biologically relevant differences in the agro-phenotypic study comparing Embrapa 5.1 and its conventional parent line. The few statistically significant differences found for the measured endpoints were not consistent across locations or across years in a particular location. Thus, these differences were not associated with a specific location nor with the gene insertion and were considered random. The study reached to the conclusion that Embrapa 5.1 is agro-phenotypically equivalent to the conventional parent line.

Likewise, analysis of compositional results revealed no biologically relevant differences. The few statistically significant differences found for certain analytes were not consistent across locations or across years in a particular location. Thus, these differences were not associated with the gene insertion. Furthermore, all values fell within the range of conventional common bean varieties with a history of safe use in Brazil. The study concluded that Embrapa 5.1 was substantially equivalent to the conventional common bean in terms of composition and nutritional value.

## Discussion and Conclusion

Based on the premise that “studies conducted in different countries may be relevant and can help risk assessors in making informed safety decisions” (Garcia-Alonso et al., 2014), recent reports support transportability showing that environmental safety conclusions from comparative assessments are consistent across geographies (Horak et al., 2015; Nakai et al., 2015; Ahmad et al., 2016; Heredia Díaz et al., 2017; Corrales Madrid et al., 2018; Clawson et al., 2019; Matsushita et al., 2020).

When a specific environmental risk hypothesis that is a particular concern for a receiving environment is identified during problem formulation, the need to consider the similarity of climatic conditions or agronomic practices could become relevant to enable transportability (Corrales Madrid et al., 2018). If after comparing environments those concerns are not addressed by the available studies, then local CFTs may be justified. Nevertheless, this would not impair transportability, as other studies will still provide data that add to the weight-of-evidence.

According to the proposed criteria for transportability of conclusions – the experimental design and methodologies, relevance and consistency of measured endpoints, and diversity of environmental conditions – the agro-phenotypic and compositional studies examined were considered appropriate for the conclusions to be transportable from Brazil to Argentina. Therefore, these conclusions could inform an eventual environmental and food / feed risk assessment of Embrapa 5.1 in Argentina.

The proposed criteria and the assessment methodology here presented, may help reduce unwarranted repeat of existing studies to conclude on environmental and/or food/feed safety of a transgenic crop. Transporting data and conclusions from CFTs can reduce the time and resources needed to conduct the risk assessment, reduce logistical and economic burdens on local, public sector and small private developers, and ultimately promote innovation by reducing unnecessary delays before beneficial technologies can be brought to market.

## Data Availability Statement

Publicly available datasets were analyzed in this study. This data can be found here: http://ctnbio.mctic.gov.br/liberacao- comercial;jsessionid=6213F402341552C281162D71DD94A906. columba?p_p_id=110_INSTANCE_SqhWdohU4BvU&p_p_life cycle=0&p_p_state=normal&p_p_mode=view&p_p_col_id= column-2&p_p_col_count=3&_110_INSTANCE_SqhWdohU4B vU_struts_action=%2Fdocument_library_display%2Fview_file_ entry&_110_INSTANCE_SqhWdohU4BvU_redirect=http%3A %2F%2Fctnbio.mctic.gov.br%2Fliberacao-comercial%2F-%2F document_library_display%2FSqhWdohU4BvU%2Fview%2F686135%3Bjsessionid%3D6213F402341552C281162D71DD94A906.columba&_110_INSTANCE_SqhWdohU4BvU_fileEntryId= 686159#/liberacao-comercial/consultar-processo.

## Author Contributions

All authors participated in the drafting of this manuscript as individual experts in their fields, and the authors are solely responsible for the contents. Any views expressed in this manuscript are the views of the authors and do not necessarily represent the views of any organization, institution, or government with which they are affiliated or employed.

## Conflict of Interest

NM was employed by Bayer Crop Science Argentina. ML was employed by Corteva Agriscience.

AM and FV declare that the research was conducted in the absence of any commercial or financial relationships that could be construed as a potential conflict of interest.

## Footnotes

1 Mercosur is the Southern common market comprising Argentina, Brazil, Paraguay and Uruguay; www.mercosur.int.

2 www.oecd.org/biotrack

## Abbreviations

CFTs: Confined field trials
ERA: environmental risk assessment
BGMV: Bean Golden Mosaic Virus.
